# Integrated micro/nano drug delivery system based on magnetically responsive phase-change droplets for ultrasound theranostics

**DOI:** 10.3389/fbioe.2024.1323056

**Published:** 2024-04-11

**Authors:** Jieying Chen, Chan Zhao, Hao Liu, Zhangchao Wang, Luyao Ma, Jiamin Zhang, Ning Xu, Ke Hu, Lei Duan

**Affiliations:** ^1^ Department of Biomedical Engineering, School of Biomedical Engineering and Informatics, Nanjing Medical University, Nanjing, China; ^2^ Department of Clinical Medical Engineering, First Affiliated Hospital of Nanjing Medical University, Nanjing, China; ^3^ Stomatological College, Nanjing Medical University, Nanjing, China; ^4^ School of Basic Medical Sciences, Nanjing Medical University, Nanjing, China

**Keywords:** phase-change droplets, microbubbles, magnetic nanoparticles, ultrasound imaging, magnetic resonance imaging, controlled drug release

## Abstract

Phase-change droplets (PCDs) are intelligent responsive micro and nanomaterials developed based on micro/nano bubbles. Subject to external energy inputs such as temperature and ultrasound, the core substance, perfluorocarbon (PFC), undergoes a phase transition from liquid to gas. This transformation precipitates alterations in the PCDs’ structure, size, ultrasound imaging capabilities, drug delivery efficiency, and other pertinent characteristics. This gives them the ability to exhibit “intelligent responses”. This study utilized lipids as the membrane shell material and perfluorohexane (PFH) as the core to prepare lipid phase-change droplets. Superparamagnetic nanoparticles (PEG-functionalized Fe_3_O_4_ nanoparticles) and the anti-tumor drug curcumin (Cur) were loaded into the membrane shell, forming magnetic drug-loaded phase-change droplets (Fe-Cur-NDs). These nanoscale phase-change droplets exhibited excellent magnetic resonance/ultrasound imaging capabilities and thermal/ultrasound-mediated drug release. The Fe-Cur-NDs showed excellent anti-tumor efficacy for the MCF-7 cells under low-intensity focused ultrasound (LIFU) guidance *in vitro*. Therefore, Fe-Cur-NDs represent a promising smart responsive theranostic integrated micro/nano drug delivery system.

## 1 Introduction

Phase-change droplets (PCDs) are a type of micro/nano material with liquid-gas phase transition capability, developed based on microbubble research (Kripfgans et al., 2000). PCDs utilize perfluorocarbons (PFCs) with low solubility and diffusion rates as the core material (Strohm et al., 2011), and use substances such as albumin (Qin et al., 2018), surfactants (Xu et al., 2018), polymers (Cai et al., 2019), lipids (Sheng et al., 2021), etc., as the membrane shell material. Drugs, proteins, targeting factors, and other substances can be further loaded inside and outside the membrane shell. Under external stimuli such as temperature, ultrasound, laser, etc., the PFC core can undergo a phase transition from liquid to gas, causing changes in PCDs’ structure, size, ultrasound imaging capability, drug release ability, and interaction with tissues/cells, thus endowing them with a more sensitive “smart responsive” ability compared to traditional microbubbles. Therefore, in recent years, PCDs have shown significant potential in research areas such as enhanced ultrasound imaging (Zhang et al., 2019), controlled drug/gene delivery (Dong et al., 2019; Sheng et al., 2021), and tumor ablation guided by high/low-intensity focused ultrasound (Li et al., 2020; Jin et al., 2021).

Magnetic iron oxide nanoparticles have long been used as contrast agents for magnetic resonance imaging (Corot et al., 2006), and they have a modifiable surface structure, which facilitates the coupling of other imaging contrast agents, drugs, and targeting factors. Moreover, under magnetic field mediation, they can also produce effects such as heating and magnetic targeting that can be utilized. Therefore, the development of theranostic integrated carrier materials based on magnetic iron oxide nanoparticles has been a research hotspot. Previous research results have shown that magnetic microbubbles formed by combining magnetic nanoparticles and microbubbles integrate the advantages of magnetic nanoparticles and microbubbles in the fields of medical imaging, molecular imaging, and drug delivery carriers. They have excellent magnetic and acoustic characteristics, and can play a positive role in applications such as multimodal imaging (Lammers et al., 2015), ultrasound/magnetic field-mediated drug delivery (Fan et al., 2013), and theranostic multimodal molecular probes (Duan et al., 2016). These achievements provide us with valuable insights: as an upgraded product of traditional microbubbles, nanoscale phase-change droplets can also be combined with magnetic nanoparticles to form magnetic phase-change droplets that possess the advantages of both. Due to the presence of a phase-changeable core, theoretically, should have more sensitive responsiveness to external energy and more precise controllability, which can achieve results in theranostic integrated applications. At present, research on magnetic phase-change droplets is still in its early stages. Existing limited studies mainly include: low-intensity focused ultrasound (LIFU)-responsive nanomedicine enables acoustic droplet vaporization-induced apoptosis of macrophages (Hou et al., 2022); Enhanced Acoustic Droplet Vaporization through the Active Magnetic Accumulation of Drug-Loaded Magnetic Particle-Encapsulated Nanodroplets (MPE-NDs) (Huang et al., 2022); Phase-transitional Fe_3_O_4_/perfluorohexane Microspheres for Magnetic Droplet Vaporization (Wang et al., 2017). Overall, there are relatively few reports on phase-change liquid carriers modified and enhanced by nanomaterials, but they hold great potential for various applications.

**FIGURE 1:**
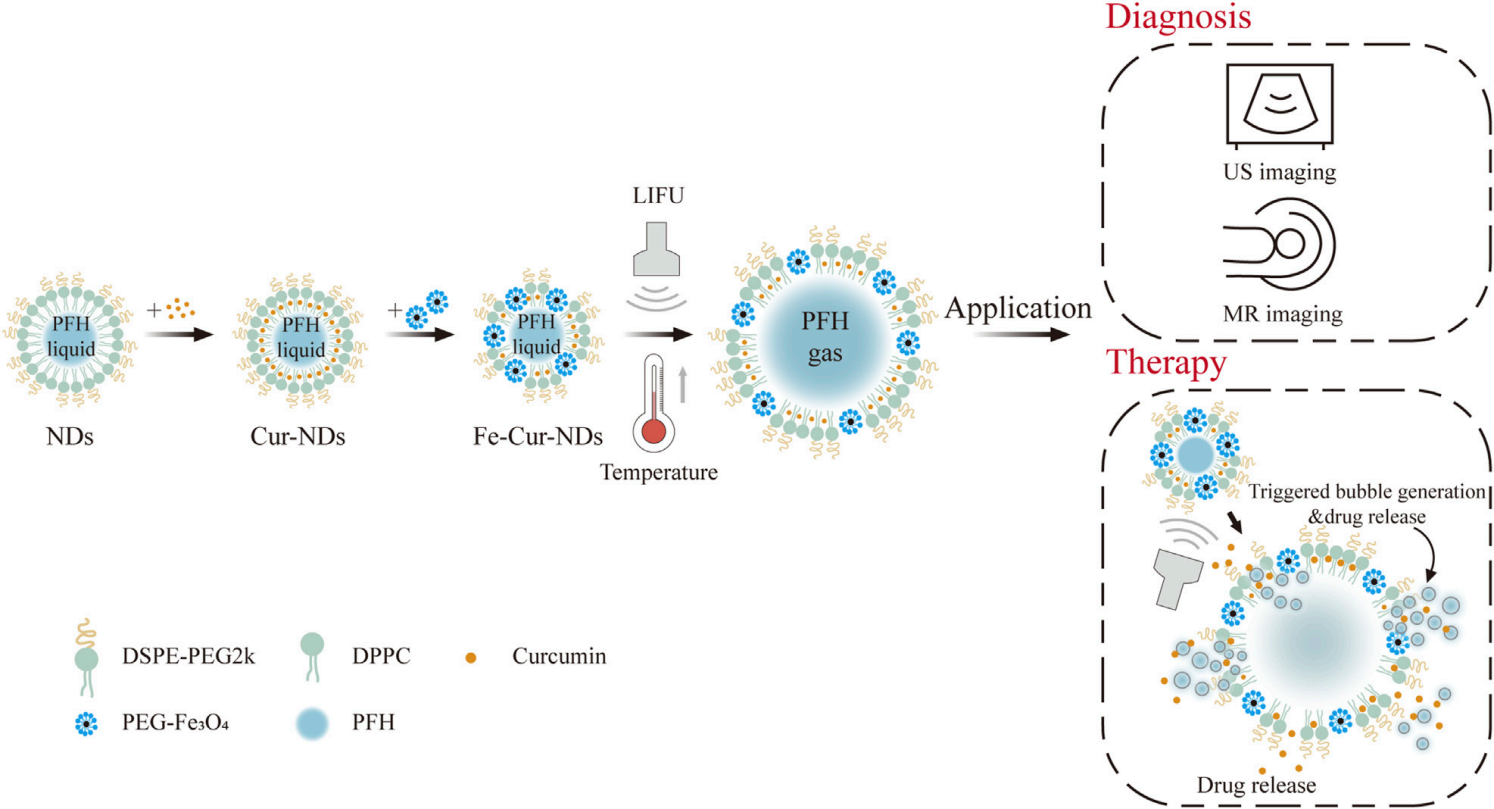
Diagram of ultrasound-responsive theranostic drug delivery system based on magnetic phase-change droplets.

Based on the background mentioned above, this paper proposes an intelligent responsive carrier with integrated diagnostic and therapeutic functions (Figure 1) based on the previous research of the research group in the field of micro/nano bubbles (Duan et al., 2016) and nano-composite materials (Zhou et al., 2017; Zhang et al., 2023a; Zhang et al., 2023b). Lipids are used as the membrane shell material, perfluorohexane (PFH) is used as the core material, and magnetic nanoparticles (PEG-functionalized Fe_3_O_4_ nanoparticles) and the anti-tumor drug curcumin are introduced into the membrane shell to construct magnetic drug-loaded phase-change droplets (Fe-Cur-NDs). The paper investigates the temperature regulation and low-intensity focused ultrasound’s control over the phase transition ability of Fe-Cur-NDs, as well as the influence of magnetic nanoparticle loading on the droplets’ phase transition ability. The paper explores the effect of Fe-Cur-NDs on *in vitro* enhanced ultrasound/magnetic resonance dual-modal imaging, as well as the potential of drug release under low-intensity focused ultrasound regulation. Through cell experiments, the safety of Fe-Cur-NDs is evaluated, and the *in vitro* inhibitory effect on breast cancer cells under ultrasound guidance is investigated.

## 2 Materials and methods

### 2.1 Materials

1,2-dipalmitoyl-sn-glycero-3-phosphocholine (DPPC), 1,2-distearoyl-sn-glycero-3-phosphoethanolamine-N-(polyethylene glycol)-2000 (DSPE-PEG(2000)), curcumin (Cur), polyethylene glycol diacrylate (PEGDA), and N,N′-methylenebisacrylamide (BIS) were purchased from Aladdin Biochemical Technology Co., Ltd. (Shanghai, China). Cholesterol (CHOL) and perfluorohexane (PFH, a fluorinated compound with a boiling point of 56 °C) were obtained from Shanghai Macklin Biochemical Technology Co., Ltd. (Shanghai, China). 5nm PEG-functionalized Fe_3_O_4_ magnetic nanoparticles (PEG-Fe_3_O_4_) were purchased from Nanjing Nanoeast Biotech Co., Ltd. (Nanjing, China). Photoinitiator (LAP) was obtained from Jiangyin Sitter Biotechnology Co., Ltd. (Jiangyin, China). Chloroform (CHCl_3_), methanol (CH_3_OH), Agar and Glycerol were purchased from Sinopharm Chemical Reagent Co., Ltd. (Shanghai, China). Dulbecco’s Modified Eagle Medium (DMEM) and phosphate-buffered saline (PBS) were obtained from Jiangsu Keygen Biotech Co., Ltd. (Nanjing, China). DAPI Staining Solution and Cell Membrane Red Fluorescent Probe (DiI) were purchased from Biyuntian Biotechnology Co., Ltd. (Shanghai, China). Penicillin-streptomycin solution (100×), CCK8 assay kit, and 4% paraformaldehyde were obtained from Biosharp Co., Ltd. (Hefei, China). Annexin V-APC/PI apoptosis kit was obtained from Elabscience Biotechnology Co., Ltd. (Wuhan, China). Trypsin-EDTA solution was purchased from Suzhou Xinsaimei Biotechnology Co., Ltd. (Suzhou, China). Fetal bovine serum (FBS) was obtained from VivaCell (Shanghai, China). MCF-7 breast cancer cells were obtained from the American Type Culture Collection (ATCC). All chemicals used were of analytical grade and required no further purification.

### 2.2 Preparation of phase-change droplets

Fe-Cur-NDs were prepared using an ultrasound emulsification method. Firstly, a mixture of 10 mg DPPC, 2 mg DSPE-PEG(2000), 2 mg CHOL, and 0.8 mg Cur was dissolved in a 10 mL CHCl_3_ solution. The solution was then transferred to a rotary evaporator (XD-52AA, Shanghai Xiande Experimental Instruments Co., Ltd., Shanghai, China) to re-move the organic solvent and form a lipid membrane at 50°C. After 2 h, 10 mL of ultrapure water (UP) was added to hydrate the lipid membrane for further use. Next, the hydrated lipid solution (10 mL) was mixed with 800 μL of PEG-Fe_3_O_4_. Then, 200 μL of PFH was added to the PEG-Fe_3_O_4_-lipid mixture and sonicated in an ice bath using an ultrasonic probe (XO-650D, Nanjing Xianou Instrument Manufacturing Co., Ltd., Nanjing, China) at a power of 130 W for 6 min (probe diameter Φ3 mm, on 2 s, off 2 s). Finally, the mixture was purified by three rounds of centrifugation (8000 rpm, 1 min 30 s, 4°C) and stored at 4°C for further use. The preparation steps for Cur-NDs, NDs, and Fe-NDs are similar to the above, except that PEG-Fe_3_O_4_ and Cur are not added, respectively. The preparation steps for Fe-DiI-NDs and DiI-NDs are also similar to the above, except replacing Cur with 50 μL 1 mM DiI.

### 2.3 The preparation of phase-change liquid droplet-hydrogel composite material

Add PEGDA, LAP, and BIS to 2 mL the Cur-NDs and Fe-Cur-NDs solutions in a molar ratio of 3.6:1.7:1. After complete dissolution, take 200 μL of the solution and place it in a circular mold with a diameter of 2 cm (Φ = 20 mm). Cure the solution using a 405 nm blue-violet light for 20 s, resulting in composite marked as Cur-NDs@Hy and Fe-Cur-NDs@Hy, respectively. The preparation steps for the water gel in the buoyancy experiment are similar to the above, except that an appropriate amount of DiI solution is added to stain the entire hydrogel.

### 2.4 Characterization of the morphology, magnetic properties, and drug loading capacity of Fe-Cur-NDs

Transmission electron microscopy (TEM) (JEM-1400Flash, JEOL, Japan) and optical microscopy (AMEX1000, Thermo Fisher, United States of America) were used to observe the morphological features of NDs. The particle size distribution and zeta potential of NDs were measured using a particle size analyzer (LitesizerTM500, Anton Paar, AUT). The magnetic properties of Fe-Cur-NDs and PEG-Fe_3_O_4_ were characterized using a vibrating sample magnetometer (VSM) (Model 7407, Lake Shore Cryotronics, United States of America). Additionally, the stability of the particle size distribution and polydispersity index (PDI) within 7 days of preparation was tested. The absorbance (Abs) of at 430 nm wavelength was measured using a UV-Vis spectrophotometer (UH5300, HITACHI, Japan) to obtain the calibration curve of Cur at different concentrations. The loading amount of Cur in Fe-Cur-NDs was determined by the absorbance of the Fe-Cur-NDs solution in CH_3_OH after ultrasonic dispersion. The encapsulation efficiency and drug loading content of Cur were calculated using the following formulas:

$$Cur\;entrapment\;efficiency\left(\%\right)=\frac{weight\;of\;Cur\;in\;NDs}{weight\;of\;Cur\;added}\times 100,$$ (1)

$$Cur\;loading\;content\left(\%\right)=\frac{weight\;of\;Cur\;in\;NDs}{weight\;of\;NDs}\times 100$$ (2)

The concentration of Fe was measured using an inductively coupled plasma-optical emission spectrometer (ICP-OES) (iCAP PRO, Thermo Fisher, United States of America).

### 2.5 Temperature/ultrasound response of Cur-NDs and Fe-Cur-NDs

The morphological effects of temperature increase on Cur-NDs and Fe-Cur-NDs were observed using an optical microscope at different temperatures: room temperature (RT), 37°C, 56°C, 65°C, and 70°C, using a constant temperature carrier platform (DB-H, Changzhou Hongze Experimental Technology Co., Ltd., China). The particle size distribution of Cur-NDs and Fe-Cur-NDs suspensions at RT, 37°C, 56°C, 65°C, and 70°C was measured using a particle size analyzer. Cur-NDs@Hy and Fe-Cur-NDs@Hy composite materials were prepared by water bath heating of phase change droplets-water gel. The microstructure of Cur-NDs@Hy and Fe-Cur-NDs@Hy was observed using an optical microscope. Low-intensity focused ultrasound (ESU-001, Shenzhen Yisu Medical Technology Co., Ltd., China) at a frequency of 840 kHz, power of 7.3 W, and a duty cycle of 1:1 was used to irradiate Cur-NDs, Fe-Cur-NDs, Cur-NDs@Hy, and Fe-Cur-NDs@Hy. The irradiation time was 0, 1, 3, 5, and 10 min. The changes in microstructure and particle size distribution of Cur-NDs and Fe-Cur-NDs after ultrasound irradiation were observed using an optical microscope and a particle size analyzer. In addition, the effect of buoyancy on Cur-NDs@Hy and Fe-Cur-NDs@Hy at 65°C for different durations was also observed.

### 2.6 *In Vitro* US Imaging/MRI of Cur-NDs and Fe-Cur-NDs

An agar phantom was prepared to simulate the acoustic ultrasonic parameters of the human tissue for ultrasound (US) imaging experiments. Agar, glycerol, and UP water were added to a beaker in the mass ratio of 3:4:90. The mixture was heated for 30–40 min until it transformed into a clear, transparent, viscous liquid. Next, the upper layer of foam was carefully removed. Then, the liquid agar was poured into a 12 × 20 × 6 cm rectangular stainless steel box. Subsequently, a 2 mL centrifuge tube was inserted to create a well-defined structure. The setup was left at room temperature until the agar completely solidified. Finally, Remove the centrifuge tube and leave a channel for adding the sample to evaluate the US imaging capabilities of NDs. The NDs (Cur-NDs and Fe-Cur-NDs) under different treatment conditions (RT, 65 °C heating for 45 s, and LIFU for 10 min) were injected into the channel of the model. Imaging of the NDs was performed at different time points using a digital ultrasound diagnostic device (C Probe, Guangzhou Sonostar Technology Co., Ltd., China) equipped with a 7.5 MHz linear array probe. For *in vitro* magnetic resonance imaging, a 7.0 T system (Biospec 7T/20 USR, Bruker, Germany) was used. Different concentrations (v/v) of Fe-Cur-NDs solution were added to a detachable 96-well culture plate with a capacity of 300 µL. To obtain T_2_ relaxation time, a multi-slice multi-echo T_2_-weighted sequence was employed. The scanning parameters were as follows: TR of 5000 m, TE of 20 m, Field of View (FOV) of 8 × 4.53 mm, matrix size of 128 × 128, and slice thickness of 2 mm. T_2_ relaxation time was calculated using the post-processing software ParaVision 6.0.1. The transverse relaxation rate (R_2_) was calculated based on the measured T_2_ data.

### 2.7 *In vitro* evaluation of drug release of Fe-Cur-NDs

To determine the effect of LIFU on the release of Cur in Cur-NDs and Fe-Cur-NDs, the NDs solution was loaded into a dialysis bag (molecular weight cut-off = 14,000 Da) and dialyzed into a 30 mL PBS solution containing 30% ethanol at 37°C with magnetic stirring at 200 rpm. The Cur-NDs and Fe-Cur-NDs solutions were divided into two groups. After 1 h and 3 h, the solutions were treated with or without LIFU (7.3 W, 10 min, 50% duty cycle). Every hour, 1 mL of dialysate was taken and mixed with an equal volume of blank solution. The concentration of Cur was then calculated by measuring the absorbance of the dialysate at 430 nm wavelength using a UV-visible spectrophotometer.

### 2.8 *In vitro* biocompatibility evaluation

For the safety evaluation of the Fe-NDs carrier material, breast cancer cells MCF-7 were seeded in a 96-well plate at a density of 4×10^4^ cells/well and allowed to adhere for 24 h. Different concentrations (10, 20, 30, 40, 50, 60, 70, 80, 90, 100 μg/mL) of Fe-NDs were co-cultured with MCF-7 cells. After 24 h of treatment, the original solution was removed, and 100 μL of fresh culture medium and 10 μL of CCK-8 solution were added to continue the incubation for 2 h. The absorbance of the cells at 450 nm wavelength was measured using a microplate reader to assess cell viability.

To assess the safety of ultrasound irradiation, breast cancer cells MCF-7 were seeded in a 96-well plate at a density of 4×10^4^ cells/well and allowed to adhere for 24 h. The bottom of the 96-well plate was coated with a coupling agent, and the treatment probe was placed at the bottom of the culture plate. Ultrasound treatment was performed using an ultrasound therapeutic device at a frequency of 840 kHz, a power of 7.3 W, a duty cycle of 1:1, and a single ultrasound irradiation time of 40, 80, 120, 160, 200, 240, 280, 320 s. After treatment, the cells were incubated for 24 h, and 10 μL of CCK-8 solution was added to continue the incubation for 2 h. The absorbance of the cells at 450 nm wavelength was measured using a microplate reader (Infinite^®^ F50, Tecan, Swiss) to assess cell viability. To determine the effect of ultrasound irradiation time on solution temperature of Water, Cur-NDs and Fe-Cur-NDs, solution temperature was measured using a thermal imager (testo 869, DEU).

### 2.9 *In vitro* evaluation of tumor cell inhibition efficacy

MCF-7 cells were seeded in a 96-well plate at a density of 4×10^4^ cells/well and allowed to adhere for 24 h. The experiment was divided into six groups: (1) Cur, (2) Cur + LIFU, (3) Cur-NDs, (4) Cur-NDs + LIFU, (5) Fe-Cur-NDs, and (6) Fe-Cur-NDs + LIFU. The concentration of curcumin was 10 μg/mL, and the ultrasound irradiation time was 120s and 200s. In the ultrasound groups, cells were co-incubated for 3 h before ultrasound treatment, followed by 21 h of further incubation. In the non-ultrasound groups, cells were directly incubated for 24 h. After incubation, 10 μL of CCK-8 solution was added to each well and further incubated for 2 h. The absorbance of the cells at 450 nm wavelength was measured using a microplate reader to assess cell viability.

To investigate the uptake of drugs in phase-change droplets by tumor cells, logarithmic phase cells were collected and seeded in a 6-well plate at a density of 1×10^5^ cells/well. After incubation at 37°C for 12 h, Cur, Cur-NDs, and Fe-Cur-NDs were added to the designated wells, with a concentration of 10 μg/mL of curcumin. After 3 h of incubation, ultrasound treatment was performed for 200s. Subsequently, cells were further incubated for 1 h and analyzed using a flow cytometer (FACS Calibur, BD Bioscience, United States of America). FlowJo10.9.0 software was used for data analysis.

For all the above experiments, all groups were set triple holes. The cells were fixed with 4% paraformaldehyde for 20 min and stained with DAPI staining solution for 5 min. PBS buffer was used for three washes. The distribution of blue fluorescence from cell nuclei and green fluorescence from curcumin was observed using an inverted fluorescence microscope (ECLIPSE Ts2R, Nikon, Japan), and the fluorescence intensity of each group was analyzed.

Apoptosis was detected by flow cytometry. MCF-7 cells in each groups were seeded in a 6-well plate at a density of 1×10^5^ cells/well. After incubation at 37°C for 12 h, Cur-NDs, and Fe-Cur-NDs were added to the designated wells, with a concentration of 10 μg/mL of curcumin. After 3 h of incubation, ultrasound treatment was performed for 200s. After 24 h of incubation, the cells were collected and resuspended in 500 μL 1x Annexin V Binding Buffer. 5 μL PI and 5 μL Annexin V-APC were added to the suspension. The cells were incubated in the dark for 15 min at room temperature and analyzed using a flow cytometer.

### 2.10 Statistical analysis

All the data were expressed as the mean ± standard deviation. The statistical data were processed with SPSS 26.0 software. The statistical significance was calculated via analysis of variance (ANOVA). A *p*-value of <0.05 was considered statistically significant (*p < 0.05, **p < 0.01).

## 3 Results and discussion

### 3.1 Morphology, magnetic properties, and drug loading characterization of phase-change droplets

**FIGURE 2:**
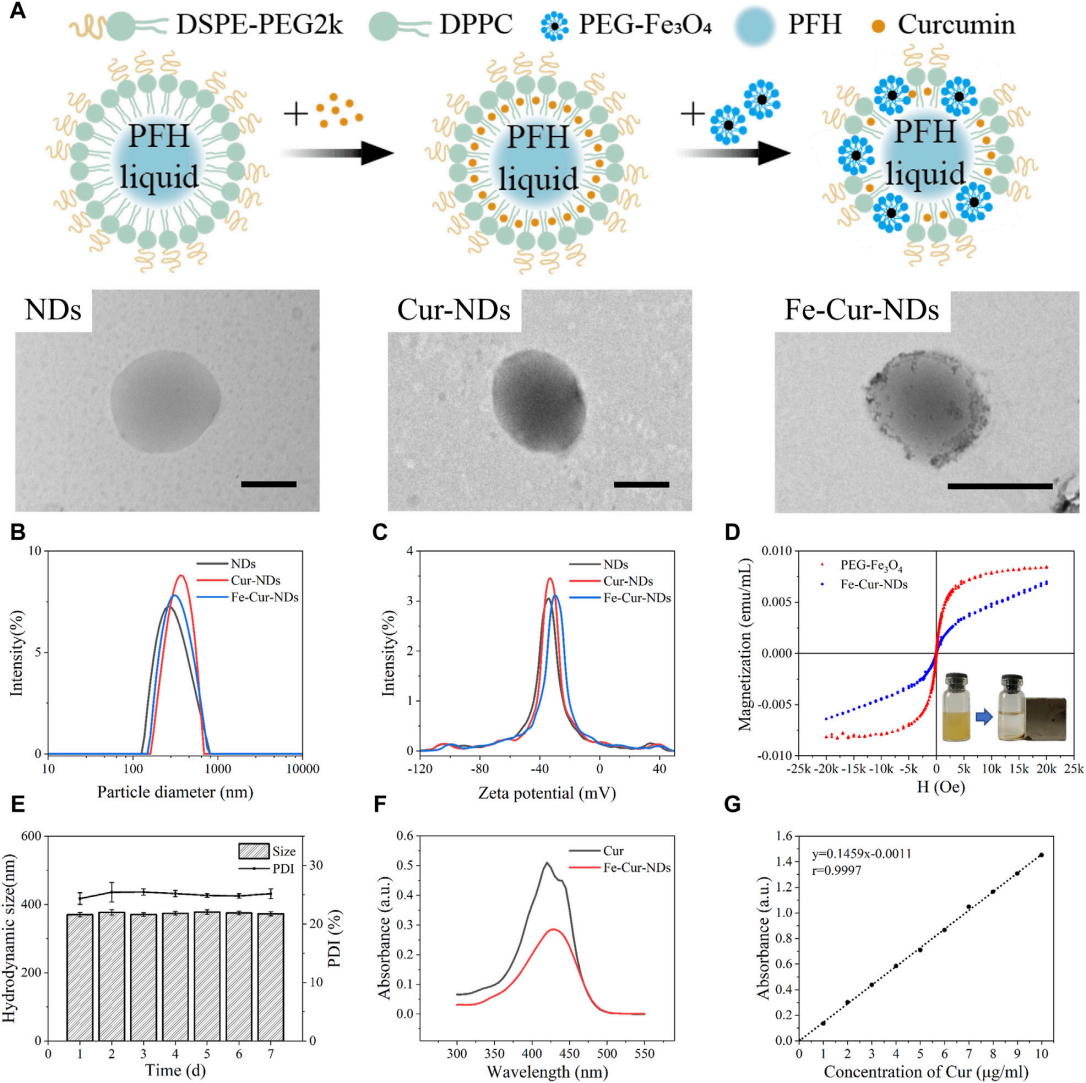
Characterization of the morphology, magnetic properties, and drug-loading performance of different types of phase-change droplets. **(A)** TEM images of NDs, Cur-NDs, and Fe-Cur-NDs (scale bar: 500 nm). **(B)** Hydrodynamic size distribution of NDs, Cur-NDs, and Fe-Cur-NDs. **(C)** Zeta potential of NDs, Cur-NDs, and Fe-Cur-NDs. **(D)** VSM curve of Fe-Cur-NDs, with an inset illustrating the magnetic responsiveness evaluation of Fe-Cur-NDs. **(E)** Changes in hydrodynamic size and PDI of Fe-Cur-NDs over 7 days. **(F)** UV-visible absorption spectra of Cur and Fe-Cur-NDs. **(G)** Standard curve of the “absorbance-centration” of Cur at a wavelength of 430 nm.

Observation of NDs, Cur-NDs, and Fe-Cur-NDs using transmission electron microscopy (TEM) (Figure 2A) revealed that they all exhibited spherical vesicular structures. In the membrane of Fe-Cur-NDs, black particle-like substances were visible, which differed significantly from the structures of NDs and Cur-NDs, confirming the effective loading of magnetic nanoparticles on the phase-change droplets. The average hydrodynamic sizes of the three types of phase-change droplets at RT (25 °C) were 321.57 ± 3.84 nm, 363.77 ± 13.11 nm, and 368.1 ± 1.42 nm, respectively (Figure 2B). The corresponding Zeta potentials were −34.77 ± 0.42 mV, −31.67 ± 1.02 mV, and −30.37 ± 0.25 mV, respectively (Figure 2C). These results indicate that all three types of phase-change droplets were in the nanoscale range before the liquid-gas phase transition, and the loading of drugs and magnetic iron oxide nanoparticles in the membrane did not significantly affect their sizes and Zeta potentials. The hydrodynamic size and PDI of Fe-Cur-NDs were monitored for seven consecutive days at 25 °C, demonstrating good size stability (Figure 2E). The iron concentration in Fe-Cur-NDs was determined to be 45.04 mg/L by ICP-OES analysis. The VSM curve of Fe-Cur-NDs (Figure 2D) indicated that they exhibited superparamagnetic behavior and possessed good magnetic targeting ability under an external magnetic field. Fe-Cur-NDs exhibited an absorption peak at 430 nm, consistent with the absorption peak of Cur, providing evidence for the loading of Cur in Fe-Cur-NDs (Figure 2F). The “absorbance-concentration” standard curve of Cur was constructed (Figure 2G), and the encapsulation efficiency of Cur in the phase-change droplets was approximately 87.09% ± 1.40%, with an average drug loading of 5.86% ± 0.18%.

### 3.2 The influence of temperature/ultrasound regulation on the phase transition ability of nanodroplets

**FIGURE 3:**
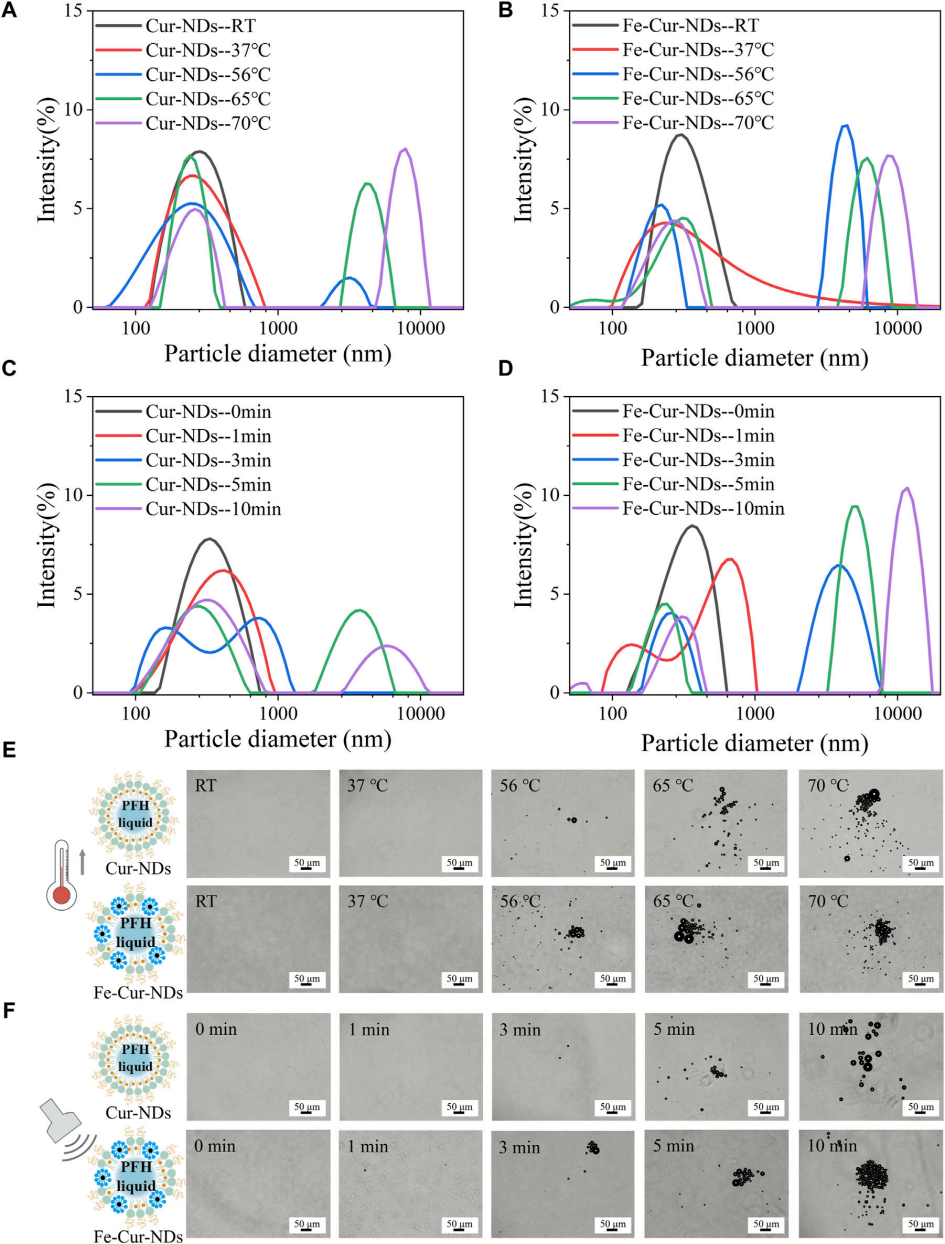
Temperature/LIFU Control of Phase-Change Droplet. Hydrodynamic size distributions of Cur-NDs **(A)** and Fe-Cur-NDs **(B)** at different temperatures (RT, 37°C, 56°C, 65°C, and 70°C). Hydrodynamic size distributions of Cur-NDs **(C)** and Fe-Cur-NDs **(D)** under low-intensity focused ultrasoundation for different durations (0, 1, 3, 5, and 10 min). **(E)** Optical microscopy images (scale bar: 50 μm) of Cur-NDs and Fe-Cur-NDs at different temperatures (RT, 37°C, 56°C, 65°C, 70°C). **(F)** Optical microscope images (scale bar: 50 μm) of Cur-NDs and Fe-Cur-NDs under low-intensity focused ultrasoundation for different durations (0, 1, 3, 5, and 10 min).

When the environmental temperature exceeds the boiling point or the pressure exceeds the vaporization threshold of the phase-change droplets’ core component, perfluorocarbon (PFC), the transition from liquid to gas can occur. PFH is a commonly used PFC with a boiling point of 56°C. It exhibits relative stability at 37°C and shows excellent repeatability in activation (Mountford et al., 2015). Therefore, PFH was used as the liquid core of the phase-change droplets in this study. Figures 3A, B show the hydrodynamic size distributions of Cur-NDs and Fe-Cur-NDs at different temperatures (RT, 37°C, 56°C, 65°C, 70°C). It can be observed that as the temperature increases, two changes occur in the hydrodynamic sizes of the two types of phase-change droplets: first, the transition from a single peak to a double peak, indicating that heating causes a partial vaporization of the core of the phase-change droplets, leading to a significant increase in particle size and the formation of a peak in the micrometer range. The remaining phase-change droplets with cores that have not undergone significant vaporization still maintain nanoscale sizes, resulting in the separation of the single peak into a double peak at RT. Second, the overall shift of the peaks to the right, as higher temperatures cause more and more nanoscale droplet cores to transition into micrometer-sized bubbles, resulting in a decrease in the amplitude of the nanoscale peak and an increase in the amplitude of the micrometer-sized peak, with the peak positions continuously shifting to the right. Figure 3E shows the morphological changes of Cur-NDs and Fe-Cur-NDs under an optical microscope as the temperature changes. It can be observed that when the temperature reaches the phase transition temperature of PFH at 56°C, micrometer-sized bubbles appear in the microscope field of view. With further temperature increase, the number and size of micrometer-sized bubbles gradually increase, visually confirming the results of the hydrodynamic size changes mentioned above. For fluorescence microscopy images studies, we synthesized Fe-DiI-NDs and DiI-NDs which were loaded Fluorescent dyes DiI to label lipids. Supplementary Figure S1A and Supplementary Figure S1B show Fe-DiI-NDs and DiI-NDs exhibited size and zeta potential characteristics similar to those of Fe-Cur-NDs and Cur-NDs. Supplementary Figure S1C better shows that there is nothing at RT, and micrometer-sized bubbles with red fluorescence can be observed on the membrane at 65°C, as well as demonstrating that the bubbles are generated by vaporization of the NDs.

Ultrasound excitation can also induce phase transition of the droplet core. Compared to the purely thermal effect caused by temperature, ultrasound can effectively stimulate droplet phase transition through a combination of cavitation, mechanical effects, and thermal effects (Rapoport et al., 2009), known as acoustic droplet vaporization (ADV). Low-intensity focused ultrasound has a higher level of safety for clinical applications and can be used as the excitation source for acoustic droplet vaporization. Figures 3C, D show the hydrodynamic size distributions of Cur-NDs and Fe-Cur-NDs under different irradiation times (0, 1, 3, 5, and 10 min) of low-intensity focused ultrasound at a frequency of 840 kHz and power of 7.3 W with a duty cycle of 1:1. Figure 3F shows the microscopic morphological changes of the droplets under an optical microscope. Supplementary Figure S1C visually displays the size changes of NDs under low-intensity focused ultrasoundation for different durations (0, 5 min) with an inverted fluorescence microscope. The results exhibit similar trends to those observed with temperature control, indicating that ultrasound excitation can induce phase transition of Cur-NDs and Fe-Cur-NDs.

Further observation of Figure 3A, Figure 3B, Figure 3C, and Figure 3D reveals distinct differences in the hydrodynamic size distributions of Cur-NDs and Fe-Cur-NDs phase-change droplets under the conditions of 37°C and 1 min of LIFU irradiation (indicated by the red curves in the figures). At 37°C, both types of droplets exhibit a single peak, but the peak width of Fe-Cur-NDs is significantly larger than that of Cur-NDs. Under 1 min of LIFU irradiation, Cur-NDs remain as a single peak, while Fe-Cur-NDs have already differentiated into a double peak, indicating that Fe-Cur-NDs have a greater number of droplets undergoing phase transition and a higher degree of phase change in the droplet cores compared to Cur-NDs under the same temperature increase or LIFU irradiation duration. This suggests that Fe-Cur-NDs are more sensitive in responding to external energy field stimulation compared to Cur-NDs.

**FIGURE 4:**
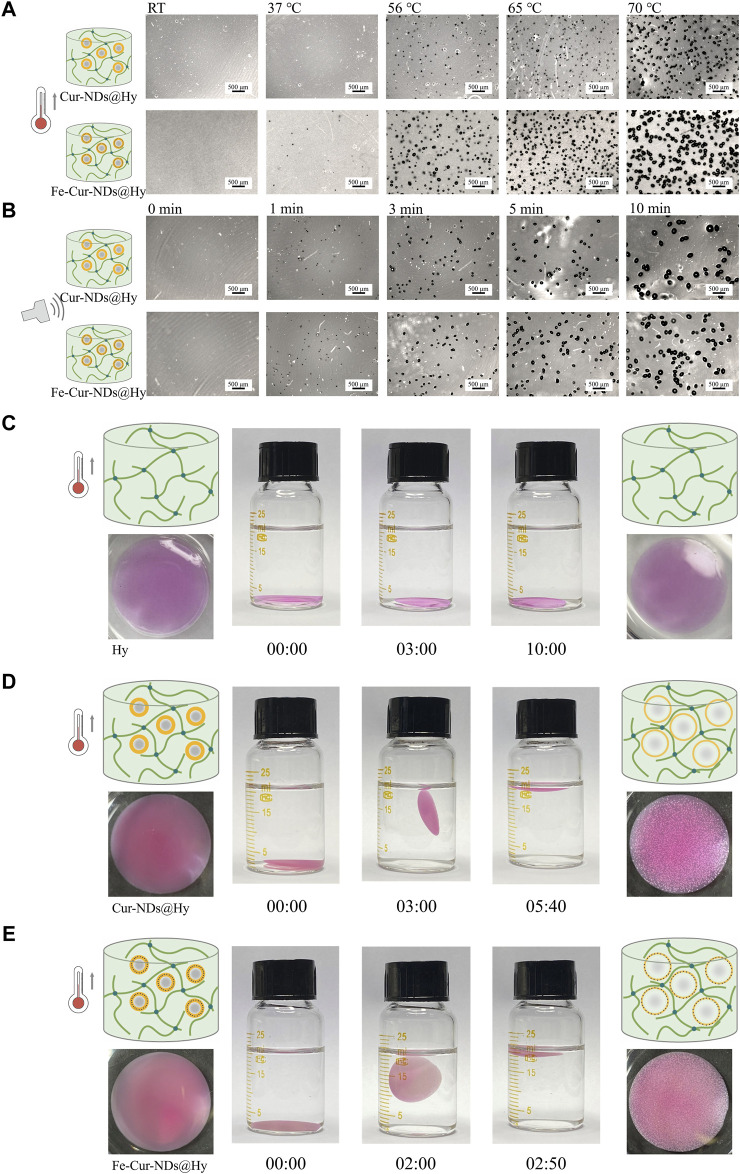
Temperature/LIFU Control of Phase-Change Droplet-Hydrogel Composite Materials Fe-Cur-NDs@Hy and Cur-NDs@Hy. **(A)** Optical microscope images (scale bar: 500 μm) of Fe-Cur-NDs@Hy and Cur-NDs@Hy at different temperatures (RT, 37 °C, 56 °C, 65 °C, 70 °C). **(B)** Optical microscope images (scale bar: 500 μm) of Fe-Cur-NDs@Hy and Cur-NDs@Hy under low-intensity focused ultrasoundation for different durations (0, 1, 3, 5, and 10 min). Macroscopic morphology and buoyancy changes of Hy **(C)**, Cur-NDs@Hy **(D)**, and Fe-Cur-NDs@Hy **(E)** under the condition of 65 °C.

To further validate and study this phenomenon, we immobilized the two types of phase-change droplets in hydrogel matrices to form Cur-NDs@Hy and Fe-Cur-NDs@Hy composite materials. By observing the changes in hydrogel structure under temperature and ultrasound control, we can objectively compare and evaluate the ability of the two types of phase-change droplets to respond to external field modulation. Figure 4A and Figure 4B show the optical microscope images of Cur-NDs@Hy and Fe-Cur-NDs@Hy at different temperatures (RT, 37°C, 56°C, 65°C, and 70°C) and under different durations of LIFU irradiation (0, 1, 3, 5, and 10 min). Macroscopic images can be found in Supplementary Figure S2. The results indicate that under temperature control or LIFU irradiation, the phase-change bubbles formed by droplet phase transition can create micrometer-sized pores inside the hydrogel. With increased external energy field strength, the number and size of pores in the hydrogel increase gradually. Under the same temperature increase or LIFU irradiation duration, Fe-Cur-NDs@Hy can generate denser and larger micropores compared to Cur-NDs@Hy, providing visual confirmation of Fe-Cur-NDs’ greater sensitivity in responding to external energy fields.

Furthermore, under the condition of 65°C, we observed and compared the buoyancy changes of the hydrogel (without phase-change droplets), Cur-NDs@Hy, and Fe-Cur-NDs@Hy in water (Figures 4C-E). The formation and structure of internal pores in the hydrogel can potentially affect its buoyancy in water. The faster and denser the formation of pores and the larger their size, the faster the hydrogel will float on the water surface. Under the condition of 65°C, the hydrogel without phase-change droplets (Hy) constantly sinks to the bottom of the container without any change in buoyancy. On the other hand, both Cur-NDs@Hy and Fe-Cur-NDs@Hy start floating at 3 min and 2 min, respectively, and remain stably floating on the liquid surface at 5 min and 40 s and 2 min and 50 s, respectively. This indicates that compared to Cur-NDs, Fe-Cur-NDs indeed exhibit a more sensitive response to external energy fields and can undergo phase transition at lower temperature and LIFU threshold, which may be attributed to the better heat absorption effect of the introduced magnetic nanoparticles in Fe-Cur-NDs (Sadeghi-Goughari et al., 2020), enhancing the thermal conversion efficiency of external energy. Additionally, previous studies have shown that the composition and structure of microbubble shells significantly influence their mechanical and transport properties. Coupling magnetic nanoparticles to the microbubble shell and altering its composition and microstructure can significantly affect their acoustic properties (Brismar et al., 2012; Duan et al., 2015). These research findings also apply to phase-change droplets. Changes in shell structure can lead to a series of changes in mechanical and transport properties and acoustic properties, ultimately affecting the efficiency of phase transition in the droplet core.

In summary, both temperature and ultrasound can control the particle size of Cur-NDs and Fe-Cur-NDs, achieving a transition from the nanoscale to the microscale. The introduction of magnetic nanoparticles can lower the temperature and LIFU threshold required for droplet phase transition, making them more sensitive to external stimulation sources. These properties endow Fe-Cur-NDs with great potential for applications in diagnosis and treatment.

### 3.3 Evaluation of enhanced ultrasound/magnetic resonance imaging effects of nanodroplets *in vitro*

**FIGURE 5:**
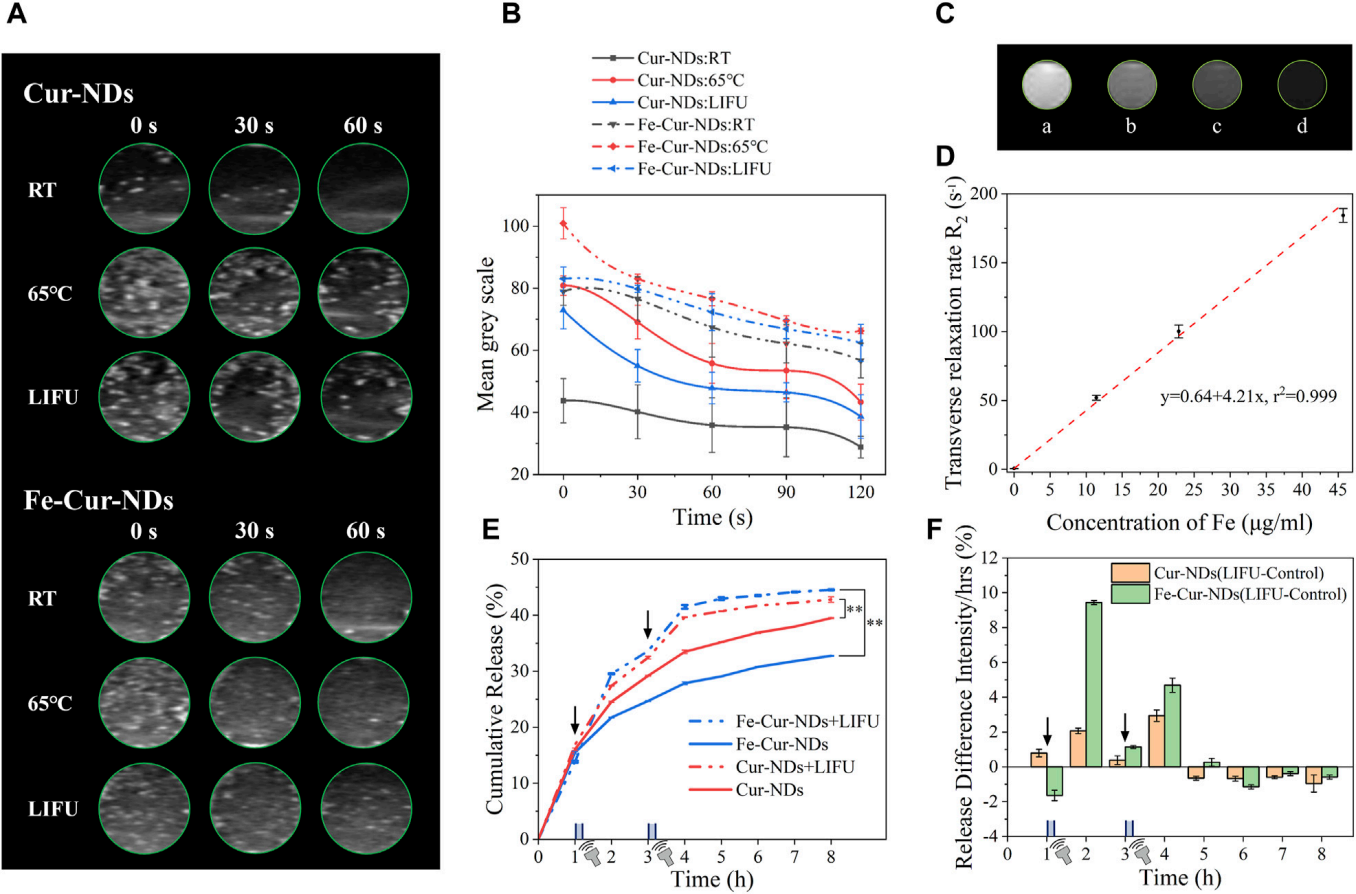
*In vitro* enhanced ultrasound/magnetic resonance imaging effects and ultrasound-regulated drug release of Fe-Cur-NDs. **(A)** Typical *in vitro* ultrasound images of Cur-NDs and Fe-Cur-NDs under RT, heating at 65 °C for 45s, and ultrasound irradiation for 10 min. **(B)** Ultrasound signal intensity-time curves of Cur-NDs and Fe-Cur-NDs. **(C)** *In vitro* MRI images of Fe-Cur-NDs (Fe concentrations in Fe-Cur-NDs: a: 0 μg/mL; b: 11.42 μg/mL; c: 22.85 μg/mL; d: 45.69 μg/mL). **(D)** Relationship between Fe content and transverse relaxation rate R_2_ in magnetic resonance imaging. **(E)** Drug release curves of Fe-Cur-NDs and Cur-NDs under conditions of no ultrasound stimulation and ultrasound stimulation (*p < 0.05, **p < 0.01). **(F)** Effect of ultrasound stimulation on drug release from Fe-Cur-NDs and Cur-NDs phase-transition droplets.

The phase transition of nanodroplets into microbubbles through liquid-gas conversion can theoretically significantly enhance their ultrasound contrast imaging capability. Figure 5A shows the *in vitro* ultrasound imaging results of Cur-NDs and Fe-Cur-NDs under temperature and ultrasound stimulation. It can be observed that both types of phase-transition droplets exhibit higher grayscale values in ultrasound images compared to before the phase transition when heated to 65 °C or stimulated with ultrasound for a certain period of time. This is because microbubbles have a larger scattering area compared to nanodroplets, and the gas core has a higher acoustic impedance than in the liquid state, thus effectively reflecting sound waves. Figure 5B displays the time-dependent curves of average signal intensity for Cur-NDs and Fe-Cur-NDs before and after the phase transition. It can be seen that the ultrasound signal intensity of both droplets is superior to the results of ultrasound stimulation after temperature stimulation. This is because temperature stimulation can uniformly and globally regulate the phase transition of the droplets compared to focused ultrasound, resulting in an overall increase in signal intensity. Additionally, regardless of the phase transition, Fe-Cur-NDs exhibit higher ultrasound signal intensity than Cur-NDs. On the one hand, this is because magnetic nanoparticles themselves have a certain enhancement effect on ultrasound imaging (Sun et al., 2019), thus increasing the baseline of Fe-Cur-NDs ultrasound imaging. On the other hand, as mentioned earlier, the introduction of magnetic nanoparticles can also lower the temperature and ultrasound threshold required for droplet phase transition, resulting in Fe-Cur-NDs having a higher number of bubbles undergoing phase transition compared to Cur-NDs under the same external energy stimulation, thereby presenting a better ultrasound imaging signal intensity than Cur-NDs.

The external stimulation to regulate the ultrasound imaging capability of phase-transition droplets partially resolves the limitations of traditional microbubbles. Before external stimulation, the perfluorocarbon (PFC) core remains in a liquid state, and the particle size of phase-transition droplets is in the nanometer range, which exhibits good circulation stability in blood vessels and can enter the lesion tissue by vessel wall dilation (Yoo et al., 2018), overcoming the clearance by the reticuloendothelial system due to the micron scale of traditional microbubbles (Barreiro et al., 2009). After external energy stimulation, the nanodroplets transform into microbubbles, overcoming the inadequate enhancement effect of nanoscale contrast agents on ultrasound signals.

Modifying magnetic nanoparticles in the membrane of phase-transition droplets not only affects their ability to respond to external energy fields, thereby impacting their acoustic properties and ultrasound imaging, but also imparts them with magnetic characteristics, which theoretically enhances magnetic resonance imaging (MRI) effects. In the experiment, Fe-Cur-NDs with Fe concentrations of 0 μg/mL, 11.42 μg/mL, 22.85 μg/mL, and 45.69 μg/mL were subjected to *in vitro* MRI imaging, and the results shown in Figure 5C. As the Fe concentration increases, the MRI images gradually darken, indicating that higher levels of magnetic nanoparticles loaded in Fe-Cur-NDs result in lower T_2_ signal intensity during MRI imaging, indicating their potential as T_2_ contrast agents. The relationship between Fe content in Fe-Cur-NDs and the transverse relaxation rate R_2_ in magnetic resonance imaging is shown in Figure 5D, demonstrating a linear relationship with a correlation coefficient of 0.999. This means that as the Fe content increases, the transverse relaxation rate R_2_ of Fe-Cur-NDs also increases. Therefore, the MRI contrast effect of Fe-Cur-NDs can be regulated by adjusting the content of magnetic nanoparticles.

### 3.4 Evaluation of *in vitro* drug release under ultrasound regulation

The prepared Cur-NDs and Fe-Cur-NDs in this study were loaded with the anti-tumor drug curcumin. Theoretically, when they respond to external temperature or ultrasound fields, the phase transition of the core in the membrane shell occurs, leading to an increase in droplet size and eventually rupture, thus promoting drug release from the membrane shell. Therefore, by adjusting the external energy field, the drug release can be regulated. However, temperature-regulated drug release through phase transition of the loaded droplets requires raising the temperature above the phase transition temperature of the core components, which is limited for *in vivo* applications. On the other hand, ultrasound has the advantages of non-invasiveness, non-radiation, and low cost, making it a hot research topic in the field of drug release for sono-dynamic therapy.

Figure 5E shows the drug release curves of the phase-transition droplets, Fe-Cur-NDs and Cur-NDs, with and without ultrasound stimulation. The results indicate that after each ultrasound stimulation, there is a significant release of drugs compared to the group without ultrasound stimulation, resulting in an increased slope of the drug release curve during the corresponding time period. This indicates that drug release from the phase-transition droplets can be controlled by low-intensity focused ultrasound stimulation. Furthermore, to investigate the effect of loading magnetic nanoparticles on drug release from the phase-transition droplets, the drug release amounts at different time points in the ultrasound stimulation group were subtracted from the corresponding time points in the group without ultrasound, resulting in a comparative graph of the effect of ultrasound stimulation on drug release from Fe-Cur-NDs and Cur-NDs (as shown in Figure 5F). It can be clearly observed that compared to Cur-NDs (orange in the graph), there is a more significant increase in drug release from Fe-Cur-NDs (green in the graph) after each ultrasound stimulation, indicating that Fe-Cur-NDs can better regulate drug release through ultrasound stimulation. This is consistent with the conclusion in Section 3.2 that the introduction of magnetic nanoparticles makes them more sensitive to external stimuli.

### 3.5 *In vitro* biocompatibility assessment of drug-loaded phase-transition droplets and their inhibitory effect on tumor cells

Prior to conducting *in vitro* tumor cell inhibition experiments, the safety and safe dosage of the carrier material should be evaluated. Supplementary Figure S3A shows the viability of breast cancer cells (MCF-7) incubated with Fe-NDs at concentrations ranging from 10 to 100 μg/mL for 24 h, as determined by CCK-8 assay. It can be observed that within the concentration range of 10–100 μg/mL, all concentrations of Fe-NDs showed minimal cytotoxicity against MCF-7 cells, with cell viability above 85%. There were no significant differences in cell viability among the different concentration groups (*p* > 0.05). This result demonstrates that the unloaded Fe-NDs have good safety profiles without significant cytotoxicity within the concentration range of 10–100 μg/mL. Therefore, for subsequent *in vitro* experiments, the concentration of the carrier material was chosen within this range.

In addition, the safety of ultrasound itself was evaluated. Under the conditions of ultrasound frequency of 840 kHz, power of 7.3 W, and a duty cycle of 1:1, the impact of ultrasound irradiation time (ranging from 40 to 320 s) on the viability of MCF-7 cells was assessed, as shown in Supplementary Figure S3B. When the ultrasound irradiation time was within the range of 40–200 s, the cell viability remained above 85%. With an extended irradiation time of 240–320 s, there was a slight decrease in cell viability but still maintained above 80%. This result indicates that short-term ultrasound treatment had no significant effect on the cells themselves. Therefore, a safe ultrasound irradiation time of within 200 s was chosen for subsequent *in vitro* cell experiments. In order to avoid the killing activities of long-term, high-power ultrasound on cells, the temperature changes of the solution at different ultrasound irradiation times were evaluated. Supplementary Figure S3C and Supplementary Figure S3D shows the temperature of all three groups increased with the extension of ultrasound irradiation time, but the temperature increase was not significant and was below 37°C within 200 s.

**FIGURE 6:**
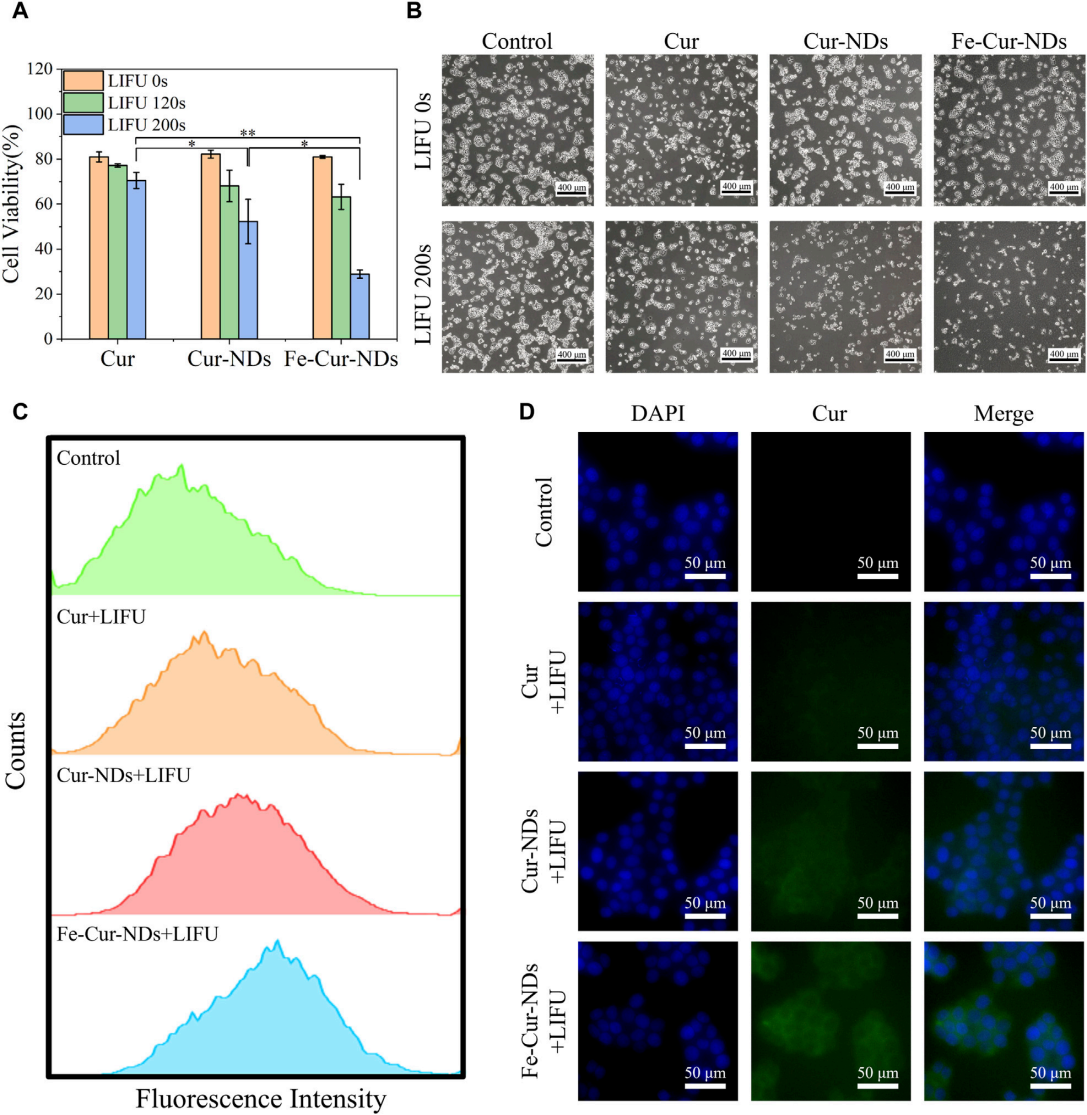
Illustrates the *in vitro* inhibitory effect of Fe-Cur-NDs on MCF-7 breast cancer cells and their uptake within tumor cells: **(A)** Inhibition of MCF-7 cells proliferation by Cur, Cur-NDs, and Fe-Cur-NDs (*p < 0.05, **p < 0.01). **(B)** Morphological comparison of MCF-7 cells with and with-out ultrasound irradiation (200 s). **(C)** Flow cytometry analysis of drug uptake by MCF-7 cells for Cur, Cur-NDs, and Fe-Cur-NDs. **(D)** Representative fluorescence microscopy images showing the drug concentration of Cur, Cur-NDs, and Fe-Cur-NDs within MCF-7 cells.

The *in vitro* proliferation inhibition effects of Cur, Cur-NDs, and Fe-Cur-NDs on MCF-7 cells are shown in Figure 6A. The results demonstrate that without low-intensity focused ultrasound stimulation, there was no significant difference in the inhibitory effects of the three groups on tumor cell proliferation, with cell viabilities around 80%. Under 120 s focused ultrasound irradiation, the cell viabilities for the three groups were 77.20%, 68.09%, and 63.18%, respectively, showing a slight decrease compared to the group without ultrasound irradiation, but without significant differences among the groups. However, when the ultrasound irradiation time was extended to 200 s, the cell viabilities for the three groups further decreased to 70.47%, 52.26%, and 28.86%, respectively, with significant differences among the groups (*p < 0.05, **p < 0.01). It can be observed that Fe-Cur-NDs exhibited a more significant inhibitory effect on tumor cell proliferation compared to the other two groups, which is consistent with the results of *in vitro* drug release mentioned earlier. This result indicates that ultrasound stimulation is a key factor in the inhibition of tumor cell proliferation by drug-loaded phase-transition droplets. Within the safe range of ultrasound irradiation time, a longer duration of ultrasound stimulation can promote rapid phase transition of the droplets, resulting in the release of a higher amount of drug components, leading to more effective inhibition of tumor cells. Figure 6B shows the morphological changes of the cells before and after ultrasound stimulation (200 s) under a microscope, which is consistent with the decrease in cell viability shown in Figure 6A.

Then, we investigate the mechanism of ultrasound-mediated inhibition of tumor cell proliferation by drug-loaded phase-transition droplets, the uptake of Cur, Cur-NDs, and Fe-Cur-NDs by MCF-7 cells was assessed under 200 s ultrasound irradiation. Curcumin emits green fluorescence, and the fluorescence intensity within MCF-7 cells was analyzed and compared using flow cytometry to analyze the differences in cellular uptake of different materials. The results are shown in Figure 6C, indicating an increasing trend in the fluorescence signal for the three groups of Cur, Cur-NDs, and Fe-Cur-NDs, with Fe-Cur-NDs demonstrating the strongest signal. Fluorescence microscopy observations of drug uptake in the three groups of cells, as shown in Figure 6D, with blue fluorescence representing the cell nucleus and green fluorescence representing curcumin, support the results obtained from flow cytometry. Compared to the Cur and Cur-NDs groups, the Fe-Cur-NDs group exhibited the highest green fluorescence signal in the cytoplasm of the cells, indicating a higher uptake rate of Fe-Cur-NDs by the cells. This result, combined with the previous findings on the inhibition of MCF-7 proliferation by Cur, Cur-NDs, and Fe-Cur-NDs (Figure 6A) and the effect of ultrasound stimulation on drug release from Fe-Cur-NDs and Cur-NDs (Figure 5F), suggests that low-intensity focused ultrasound can regulate the release of effective drug components loaded in the phase-transition droplets by promoting phase transition of the droplets’ core. Moreover, Fe-Cur-NDs, which incorporate magnetic nanoparticles and exhibit a more sensitive response to ultrasound stimulation, can release drugs more efficiently. Furthermore, ultrasound-induced biological effects such as sonoporation and localized hyperthermia, resulting from the cavitation and heating of microbubbles formed after the phase transition of the droplets, can enhance the permeability of the tumor cell membrane, improve drug diffusion, and enhance drug delivery efficiency in the tumor site (Duan et al., 2020). As a result, more drug components released from the phase-transition droplets can be taken up by the cells, leading to better inhibition of tumor cell growth.

**FIGURE 7:**
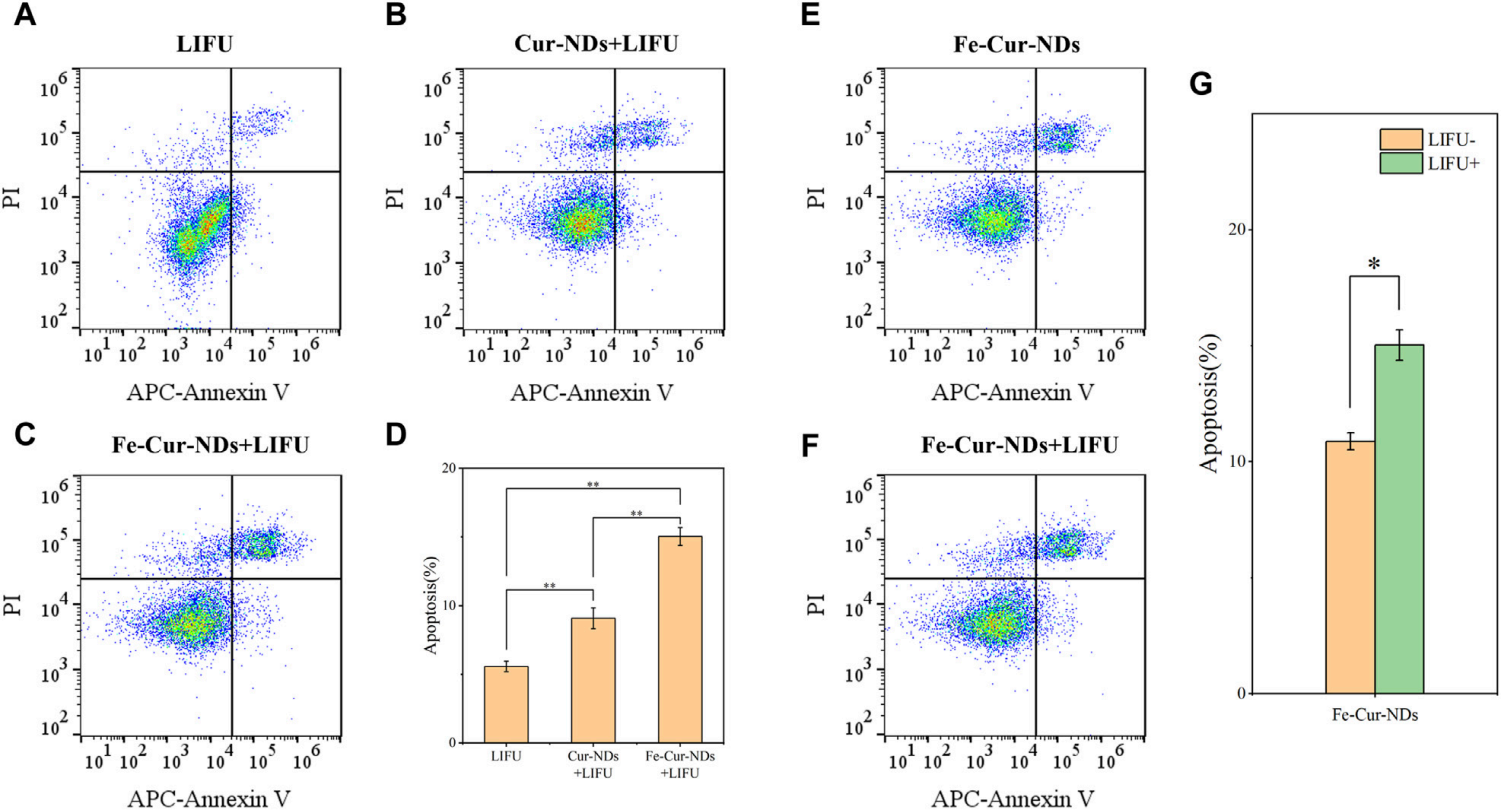
Induction of apoptosis in MCF-7 breast cancer cells by Fe-Cur-NDs: Flow cytometry was used to assess the effects of **(A)** LIFU, **(B)** Cur-NDs + LIFU, and **(C)** Fe-Cur-NDs + LIFU on MCF-7 cell apoptosis. **(D)** Apoptosis rates of MCF-7 cells induced by the LIFU group, Cur-NDs + LIFU, and Fe-Cur-NDs + LIFU groups (*p < 0.05, **p < 0.01). Effect of Fe-Cur-NDs on MCF-7 cell apoptosis **(E)** without ultrasound irradiation and **(F)** with ultrasound irradiation. **(G)** Apoptosis rates of MCF-7 cells induced by Fe-Cur-NDs with and without ultrasound irradiation.

To further study the biological effects of the carrier material, the impact on tumor cell proliferation was evaluated using bromodeoxyuridine (BrdU) immunofluorescent staining, with results presented in Supplementary Figure S4. Compared to the LIFU group, both Fe-Cur-NDs + LIFU and Cur-NDs + LIFU groups significantly inhibited the *in vitro* proliferation of MCF-7 tumor cells, with Fe-Cur-NDs under ultrasound irradiation conditions exhibiting a more pronounced inhibitory effect on cell proliferation than Cur-NDs. Under ultrasound irradiation conditions, the induction of apoptosis in MCF-7 was detected using Annexin V-APC/PI flow cytometry. Results showed that Fe-Cur-NDs significantly induced apoptosis in tumor cells (Figures 7A–D). Additionally, the effect of Fe-Cur-NDs on tumor cell apoptosis with and without ultrasound irradiation was observed. Compared to the treatment group without ultrasound irradiation, Fe-Cur-NDs significantly enhanced the apoptosis rate of MCF-7 under ultrasound irradiation (Figures 7E–G).

## 4 Conclusion

This study introduces magnetic nanoparticles and anti-tumor drugs into the membrane shell of nanoscale phase-transition droplets, creating Fe-Cur-NDs. Fe-Cur-NDs can sensitively respond to external temperature/ultrasound stimulation, achieving size modulation from the nanoscale to the micrometer scale through the phase transition of the inner core liquid-gas phase. This effectively enhances ultrasound imaging and magnetic resonance imaging. Additionally, the structural changes in the droplet membrane shell caused by the liquid-gas phase transition promote the release of effective drug components, enabling controlled and efficient drug delivery under ultrasound regulation. *In vitro* cell experiments confirmed the safety of Fe-Cur-NDs and demonstrated that they exhibit more effective inhibition of breast cancer cell growth under ultrasound stimulation compared to Cur-NDs without magnetic nanoparticles. Furthermore, the introduction of magnetic materials also provides Fe-Cur-NDs with the potential for further investigation of therapeutic functions such as magnetic targeting and magnetic hyperthermia. Therefore, Fe-Cur-NDs represent a promising smart, responsive, and integrated micro/nano drug delivery system for diagnostics and therapy.

## Data Availability

The raw data supporting the conclusion of this article will be made available by the authors, without undue reservation.
