# Supplementary material for: Integrated micro/nano drug delivery system based on magnetically responsive phase-change droplets for ultrasound theranostics
Source: Front Bioeng Biotechnol. 2024 Apr 11;12:1323056. doi: 10.3389/fbioe.2024.1323056 (PMC11043469; doi:10.3389/fbioe.2024.1323056)
Supplement: Supplementary file 1 [file DataSheet1.docx]

Supplementary Material

Integrated micro/nano drug delivery system based on magnetically responsive phase-change droplets for ultrasound theranostics


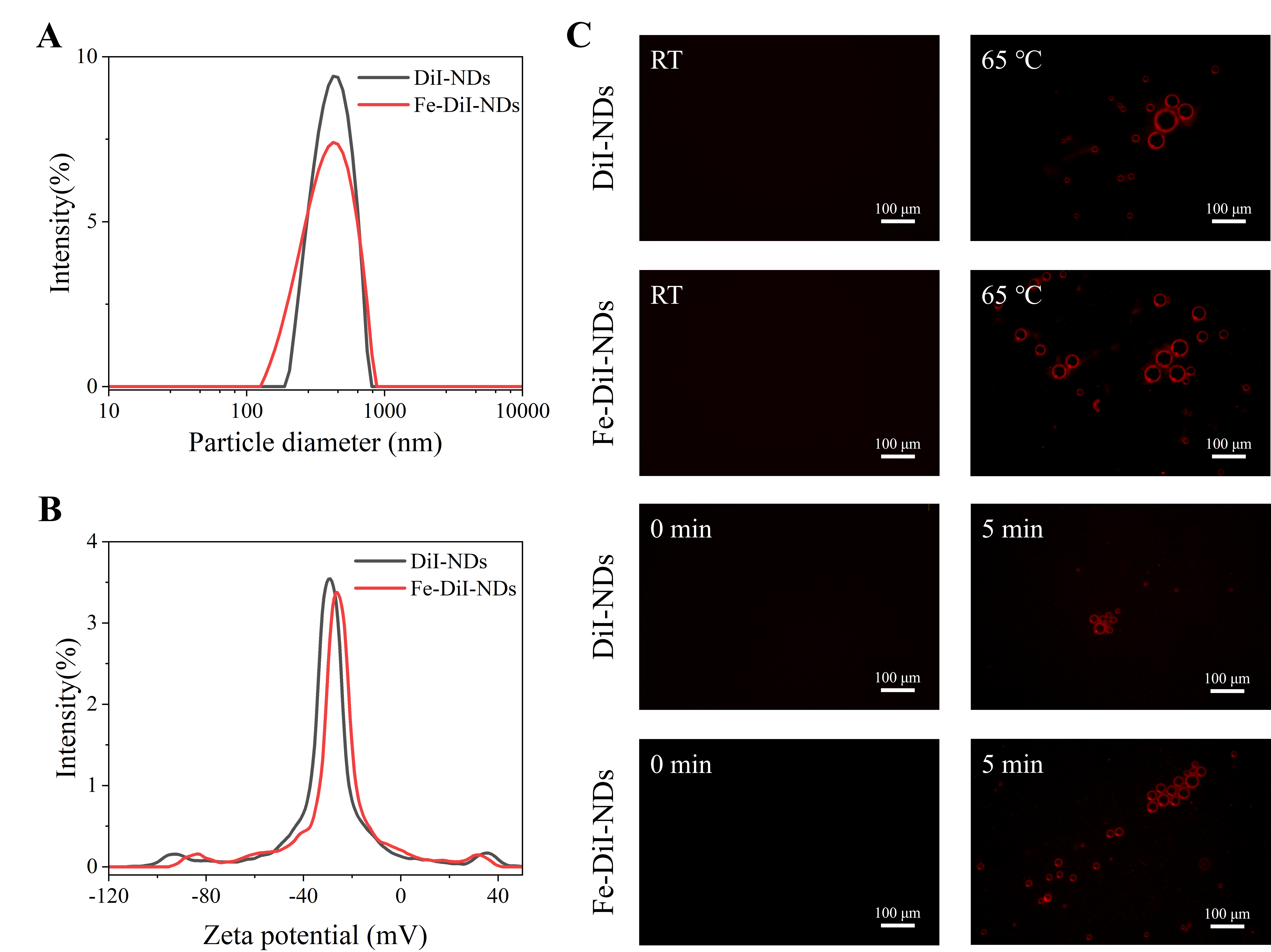


**Figure S1.** Characterization and Temperature/LIFU Control of Fe-DiI-NDs and DiI-NDs. (**A**) Hydrodynamic size distribution of Fe-DiI-NDs and DiI-NDs. (**B**) Zeta potential of Fe-DiI-NDs and DiI-NDs. (**C**) Fluorescence microscopy images (scale bar: 100 μm) of Fe-DiI-NDs and DiI-NDs at different temperatures (RT, 65°C) and under low-intensity focused ultrasoundation for different durations (0, 5 minutes).


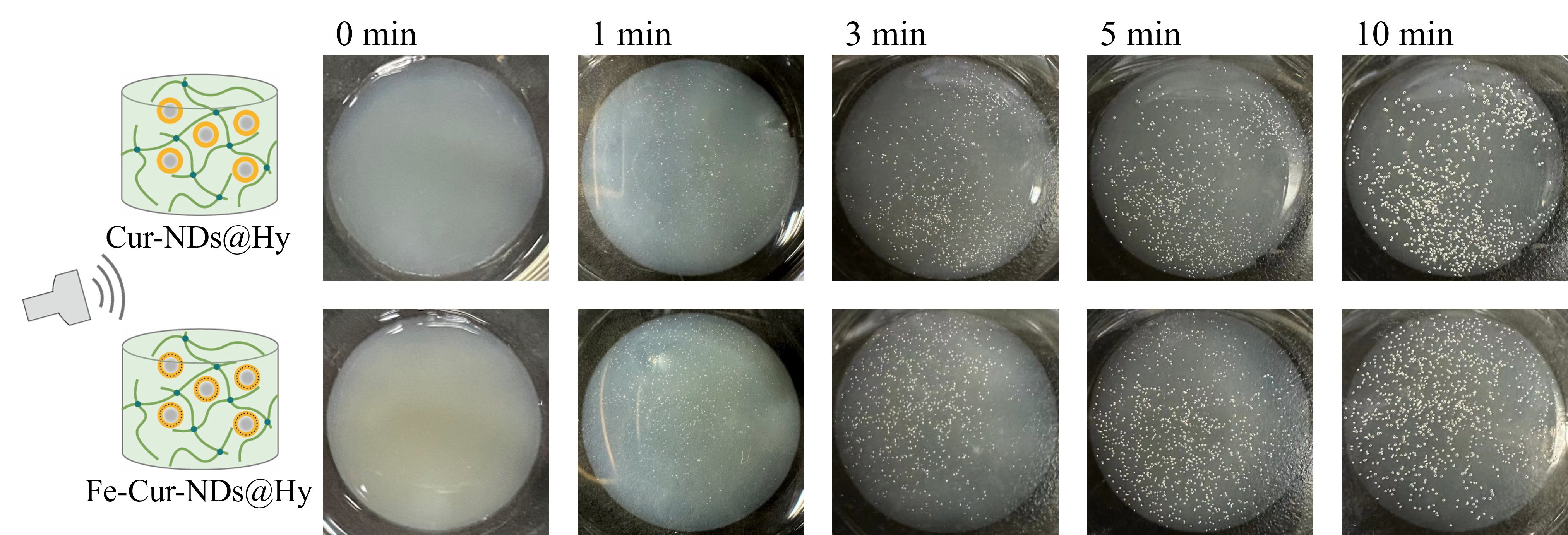


**Figure S2.** LIFU Control of Phase-Change Droplet-Hydrogel Composite Materials Fe-Cur-NDs@Hy and Cur-NDs@Hy. Macroscopic images (hydrogels are Φ20 mm circles) of Fe-Cur-NDs@Hy and Cur-NDs@Hy under low-intensity focused ultrasoundation for different durations (0, 1, 3, 5, and 10 minutes).


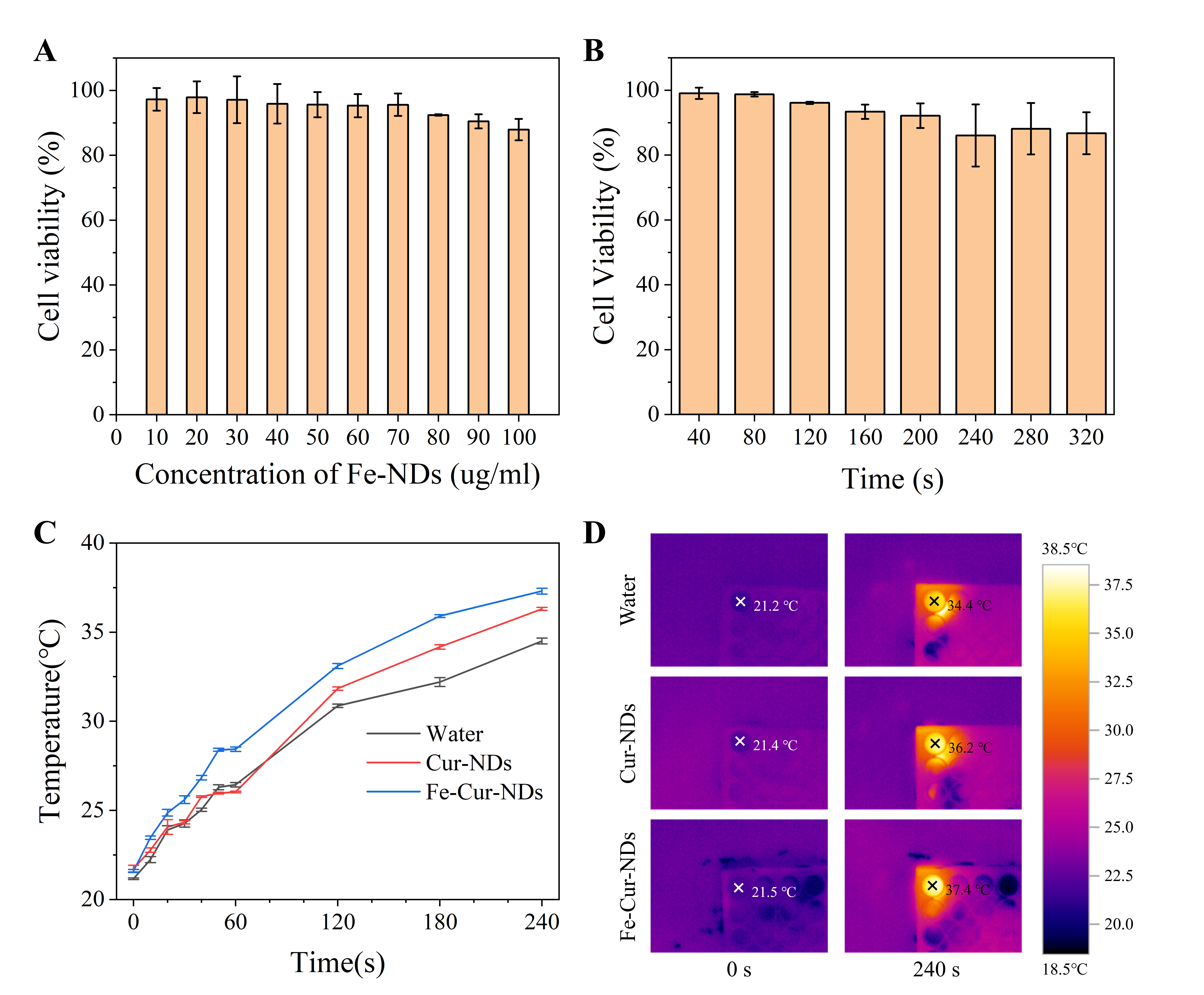


**Figure S3.** In vitro safety assessment of carrier materials and ultrasound irradiation time. (**A**) The impact of concentrations of Fe-NDs on the viability of breast cancer cells (MCF-7). (**B**) The impact of ultrasound irradiation time on the viability of breast cancer cells (MCF-7). (**C**) Solution temperature curves of water, Fe-Cur-NDs and Cur-NDs under different ultrasound irradiation time. (**D**) Typical thermal images of water, Cur-NDs and Fe-Cur-NDs under ultrasound irradiation for 0 and 240 seconds.


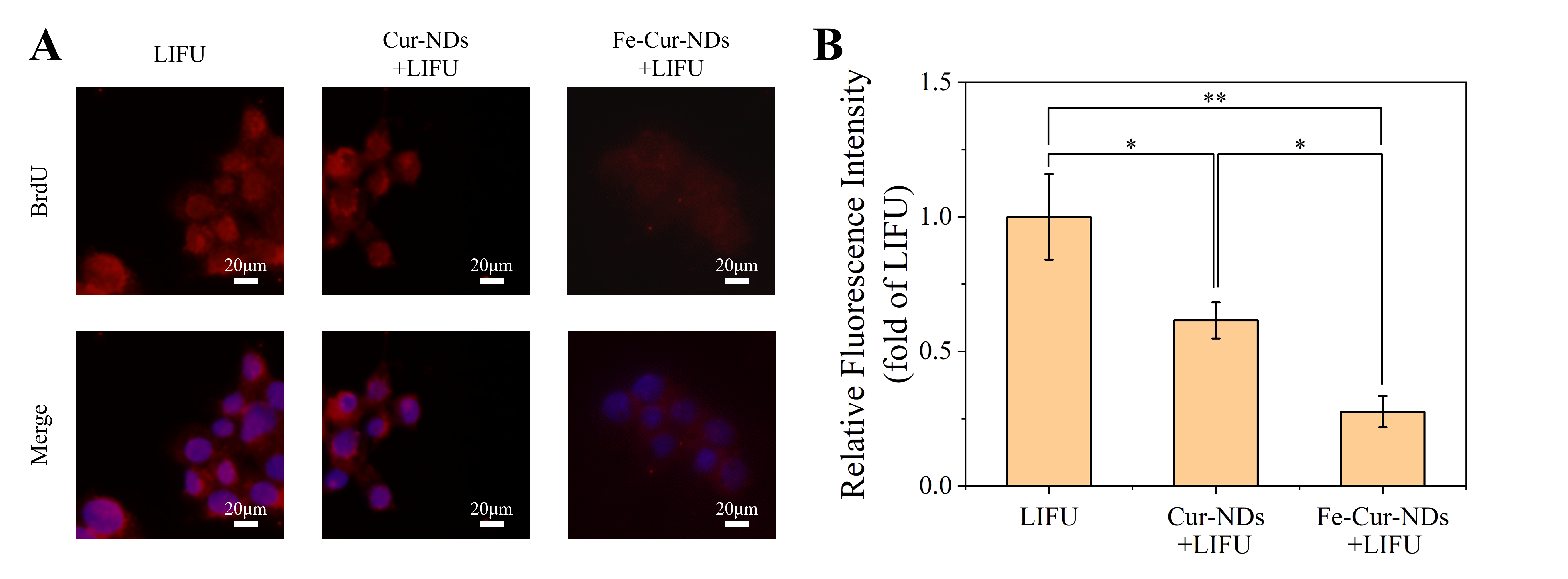


**Figure S4.** Effect of Fe-Cur-NDs on the proliferative capacity of MCF-7 breast cancer cells. (A) Representative fluorescence microscopy images of BrdU immunofluorescent staining within MCF-7 cells among the LIFU group, Cur-NDs+LIFU, and Fe-Cur-NDs+LIFU groups. (B) Compared to the LIFU group, the relative average fluorescence intensity of BrdU within MCF-7 cells in the Cur-NDs+LIFU and Fe-Cur-NDs+LIFU groups (*P＜0.05, **P＜0.01).
